# Pre‐treatment analysis of non‐rigid variations can assist robust intensity‐modulated proton therapy plan selection for head and neck patients

**DOI:** 10.1002/mp.15971

**Published:** 2022-10-06

**Authors:** Ying Zhang, Jailan Alshaikhi, Richard A. Amos, Wenyong Tan, Virginia Marin Anaya, Yaru Pang, Gary Royle, Esther Bär

**Affiliations:** ^1^ Department of Medical Physics and Biomedical Engineering University College London Gower Street London UK; ^2^ Saudi Proton Therapy Center King Fahad Medical City Riyadh Saudi Arabia; ^3^ Department of Oncology Shenzhen Hospital of Southern Medical University Shenzhen Guangdong China; ^4^ University College London Hospitals NHS Foundation Trust Radiotherapy Physics London UK

**Keywords:** anatomical uncertainty, head and neck cancer, intensity‐modulated proton therapy, robust evaluation

## Abstract

**Purpose:**

To incorporate small non‐rigid variations of head and neck patients into the robust evaluation of intensity‐modulated proton therapy (IMPT) for the selection of robust treatment plans.

**Methods:**

A cohort of 20 nasopharynx cancer patients with weekly kilovoltage CT (kVCT) and 15 oropharynx cancer patients with weekly cone‐beam CT (CBCT) were retrospectively included. Anatomical variations between week 0/week 1 of treatment were acquired using deformable image registration (DIR) for all 35 patients and then applied to the planning CT of four patients who have kVCT scanned each week to simulate potential small non‐rigid variations (sNRVs). The robust evaluations were conducted on IMPT plans with: (1) different number of beam fields from 3‐field to 5‐field; (2) different beam angles. The robust evaluation before treatment, including the sNRVs and setup uncertainty, referred to as sNRV+R evaluation was compared with the conventional evaluation (without sNRVs) in terms of robustness consistency with the gold standard evaluation based on weekly CT.

**Results:**

Among four patients (490 scenarios), we observed a maximum difference in the sNRV+R evaluation to the nominal dose of: 9.37% dose degradation on *D*_95_ of clinical target volumes (CTVs), increase in mean dose (*D* ${}_{\text{mean}}$) of parotid 11.87 Gy, increase in max dose (*D* ${}_{\max}$) of brainstem 20.82 Gy. In contrast, in conventional evaluation, we observed a maximum difference to the nominal dose of: 7.58% dose degradation on *D*_95_ of the CTVs, increase in parotid D${}_{\text{mean}}$ by 4.88 Gy, increase in brainstem *D* ${}_{\max}$ by 13.5 Gy. In the measurement of the robustness ranking consistency with the gold standard evaluation, the sNRV+R evaluation was better or equal to the conventional evaluation in 77% of cases, particularly, better on spinal cord, parotid glands, and low‐risk CTV.

**Conclusion:**

This study demonstrated the additional dose discrepancy that sNRVs can make. The inclusion of sNRVs can be beneficial to robust evaluation, providing information on clinical uncertainties additional to the conventional rigid isocenter shift.

## 1 INTRODUCTION

Intensity‐modulated proton therapy (IMPT) offers the potential to limit dose to normal tissues for head and neck (H&N) cancer patients. However, anatomical variations in the radiation area increase dosimetric uncertainty during treatment delivery.^7^, ^8^ Progressive changes due to weight loss, tumour shrinkage, and parotid glands shrinkage were reported as 3.9%–25.5% (weight loss), 20%–60% (tumor shrinkage), and 21.3%–42% (parotid glands shrinkage).^9^, ^10^, ^11^ Neck folds, neck tilts, spine flexions, and jaw and shoulder position changes also commonly occur during the H&N cancer treatment.^12^, ^13^ These small non‐rigid variations (sNRVs) cannot be simplified as rigid translations and, unlike progressive changes that are patient‐specific, sNRVs occur randomly.

Current research in H&N proton therapy focuses on the development of adaptive strategies to mitigate the influence of progressive anatomical changes. In clinical practice, offline adaptive planning strategies are applied when a threshold of dose to a critical structure is reached.^14^, ^15^ This method is effective, but delays in implementing adaptive re‐plans exist due to time required for imaging, re‐planning, plan approval, and plan verification. This reactive approach to adaptive therapy poses workflow challenges for the busy clinical practice. To mitigate time delay during the offline adaptive process, the use of anatomical modeling was suggested.^16^, ^17^ Anatomical models can accurately predict the patients' progressive changes and can therefore be used to create adaptive plans in advance, which can be applied as soon as the adaption threshold is reached. Online adaption is intended for same‐day application. However, due to computational limitations, online adaption either compromises on accuracy or constrains the optimizer. Matter et al.^18^ used an analytical pencil beam algorithm to generate plans in 10 s. However, analytical calculations overestimate the target by 10% and underestimate some organs at risk (OARs) by up to 10 Gy.^19^ Bobić et al.^20^ constrained the optimizer to adjust the beamlet positions, energies, and beamlet weights to produce adapted plans. They reported a median adjustment time of 12 min excluding the time taken for deformable image registration (DIR). Lalonde et al.^21^ only adjusted the weights of the beamlets to produce adapted plans, their median adjustment time was also 12 min but included the time for DIR. When plans are adapted either online or offline, the patient position may be different from the position in the image. sNRVs not captured during imaging will still be present.

In addition to adaptive planning strategies that mitigate the dosimetric impact of anatomical variability, evaluation of plan robustness is also used.^22^, ^23^ Set up and range uncertainty are considered in conventional robust evaluation. Treatment plan evaluation including inter‐fractional anatomical variations often uses images acquired during the treatment,^24^, ^25^, ^26^ and as such, they can only inform the planning process for a portion of the treatment delivery. A more complete robust evaluation including the possible sNRVs before treatment is crucial to design a plan that is robust toward these anatomical changes. Because sNRVs are not patient‐specific, they can be included into robust evaluation to provide additional information before treatment. To our knowledge, studies have yet to reveal the dosimetric impact of sNRVs on proton therapy plans.

Range uncertainty in robust analysis evaluates the dosimetric impact of the systematic uncertainty in calculated range based on CT calibration and conversion to relative stopping power (RSP), while setup uncertainty reflects random errors throughout a course of therapy. This study focused on random errors. We aim to (1) establish the additional impact of sNRV, as a component of random setup error, over and above the rigid translation by building a distribution of possible sNRVs based on population data; (2) provide a robust evaluation method based on the probability distribution. The benefit of this new evaluation method was compared to the conventional robust evaluation, with gold‐standard evaluation (after‐treatment evaluation that used weekly repeated CTs) as the reference for quantification.

## 2 METHODS

### 2.1 Patient data

Twenty nasopharynx cancer patients with weekly repeat CT and 15 oropharynx cancer patients with weekly cone‐beam CT (CBCT) who received photon therapy were recruited retrospectively. We obtained deformations between week 0 (planning CT) and week 1 of treatment (the time between planning CT and treatment week 1 is 14 days, which is the standard time for treatment planning) for all 35 patients, creating a distribution of possible sNRVs based on the method described in Section 2.2. Examples of sNRVs are shown in Appendix A. Four nasopharynx patients who have weekly repeat CTs were randomly selected as test dataset, where we applied the 35 sNRVs to their planning CT.

We evaluated the robustness of IMPT plans toward the uncertainty (see Section 2.3) applied to the test patients. We evaluated the following scenarios: (1) different number of fields from 3‐field to 5‐field plan; (2) different beam angles. The different beam arrangements used in this paper are listed in the upper part of Table 1 and illustrated in Appendix B. The targets (both tumor and nodal area) were split for different fields in these IMPT plans. All plans were robustly optimized using ±3 mm setup and ±3.5% range uncertainty in Eclipse version 16.1.0 (Varian Medical Systems, Palo Alto, CA). A relative biological effectiveness (RBE) of 1.1 for proton beams was used. The dosimetric goals for all plans in this study are summarized in the lower part of Table 1. A plan was deemed robust (stop optimization) if the goals set for the clinical target volumes (CTVs) and serial organs are fulfilled for all 12 dose distributions (3 mm orthogonal shifts combined with the ±3.5% range error) as well as the nominal scenario.

**TABLE 1:** Plan beam arrangements and dosimetric goals used in this paper

Plan beam arrangements	
Beam arrangements	Angle
3*B*_45_	45 180 315
3*B*_60_	60 180 300
4*B*_110_	60 110 250 300
4*B*_120_	60 120 240 300
5*B*	60 110 180 250 300

Dosimetric goals of the treatment plans	
Structure	Metric goal under uncertainty		Robust optimized
High‐risk CTV	*D*_95_	*>* 95% of prescription dose (72.6Gy)	Yes
Low‐risk CTV	*D*_95_	*>* 95% of prescription dose (63Gy)	Yes
CTV	*D*_2_	*<* 107% of prescription dose	Yes
Spinal cord	*D*_max_	*<*45 Gy	Yes
Brainstem	*D*_max_	*<*55 Gy	Yes
Chiasm	*D*_max_	*<*55 Gy	Yes
Optical Nerve	*D*_max_	*<*55 Gy	Yes
Structure	Metric goal in nominal		
Parotid glands	*D*_mean_	*<*26 Gy	Yes
Cochlea	*D*_mean_	*<*45 Gy	Yes
Oral cavity	*D*_mean_	*<*40 Gy	No
Larynx	*D*_mean_	*<*40 Gy	No

Proton planning information: MFO planning; spot spacing size: 5 mm; energy range:

70–250 MeV; range shifter: 5 cm; dose calculation algorithm: pencil beam algorithm (PBA); optimization algorithm: nonlinear universal proton optimizer.

### 2.2 Extracting small non‐rigid variations from CT images

Anatomical variations during the first week of treatment are predominately due to sNRVs, whereas progressive changes (weight‐loss, tumor shrinkage) are less significant.^27^, ^28^, ^29^ Thus, the anatomical changes in the first week from a cohort of patients can be seen representative of a distribution of possible sNRVs.

The sNRVs of a cohort of patients (see Section 2.1) were captured using DIR. DIR finds the optimal deformation vector field (DVF) ϕ to achieve the greatest similarity between two images. We used stationary velocity fields (SVFs) v of diffeomorphic image registration to identify anatomical changes in this project. SVFs can easily be calculated from the inverse DVFs ϕ using:^30^

(1) $$\phi =\exp\left(v\right)\;\Rightarrow \;\phi^{-1}\left(x\right)=\exp\left(-v\right).$$

To apply the deformations between groups of subjects, we need to project the SVFs into the atlas space, in which all the SVFs have the same position and resolution. The atlas was obtained from a group‐wise registration which spatially normalized a cohort of patients^[1]^ 1.^16^, ^31^ In the procedure of the projection, the planning CT (pCT) of each patient was the reference geometry, and the CT acquired during the first treatment week (CT_*t*_) was registered to the pCT to produce $v_{p\to t}$, where *p* stands for pCT and *t* stands for the week (in this case t=1) when the weekly CT acquired. Then, each patient's pCT was registered to the atlas to produce $v_{a\to p}$, where *a* stands for atlas. $v_{a\to p}$ transformed the inter‐patient velocity fields $v_{p\to t}$ into the atlas using

(2) $$v_{a,p\to t}=v_{a\to p}^{-1}\circ v_{p\to t}\circ v_{a\to p},\;p\forall P.$$

*P* includes all the patients' data used in this study.

Then $v_{a,p\to t}$ was transformed into the space of an individual patient $\tilde{p}$ using

(3) $$v_{\tilde{p}\to t}\approx v_{a\to \tilde{p}}^{-1}\circ v_{a,p\to t}\circ v_{a\to \tilde{p}}.\;p\forall P.$$

The deformation $v_{\tilde{p}\to t}$ was used for warping pCT to simulate an sNRV. Finally, in order to warp the planning image $I_{\tilde{p}}$, the transformation must be directed from the predicted anatomy to the pCT. This can be simply achieved by reversing the SVFs using

(4) $$v_{t\to \tilde{p}}=-v_{\tilde{p}\to t}.$$

The warped image CT${}^{\text{sNRV}}$ was acquired from:

(5) $$\phi_{t\to \tilde{p}}=\text{exp}\left(v_{t\to \tilde{p}}\right),$$

(6) $${\text{CT}}_{\tilde{p}}^{\text{sNRV}}=\phi_{t\to \tilde{p}}\left(\text{pCT}\right),$$

with *t* = 1 for all the equations above. This method produced 35 CT${}^{\text{sNRV}}$s for each patient to represent the possible sNRVs.

The diffeomorphic image registration is implemented in NiftyReg^[1]^. NiftyReg is an open‐source DIR tool available as part of the NifTK project.

### 2.3 Robustness evaluation

We included the 35 sNRV scenarios of each test patient into the robustness evaluation using CT${}^{\text{sNRV}}$s. For the four test patients, the dose distributions of IMPT plans were calculated under each robustness scenario. We compared (1) the robust evaluation based on the sNRV scenarios and rigid translation with (2) the conventional setup setting that only includes rigid translation. Probability analysis was used in these two before‐treatment evaluations to rank the robustness of IMPT plans for each robustly optimized dose metric listed in the lower part of Table 1.

#### 2.3.1 Robustness evaluation scenarios

For our proposed evaluation method using the sNRV scenarios (1), we simulated the isocenter shift for each cardinal direction (*x*_*n*_,*y*_*n*_,*z*_*n*_) following the Gaussian distribution with mean μ = 0 mm and standard deviation σ = 1.5 mm^25^ on the 35 CT${}^{\text{sNRV}}$s. This was done to calculate the perturbed dose distributions caused by the sNRVs and rigid setup uncertainty, since the CT${}^{\text{sNRV}}$s have the same isocenter as the planning CT. The 35 dose distributions for each IMPT plan were included in this sNRV+R evaluation.

The conventional evaluation (2) only include the rigid setup uncertainty by applying the same isocenter shifts used in the sNRV+R evaluation to the planning CT. This way, we achieved 35 perturbed dose distributions per IMPT plan, which were included to evaluate the plan robustness.

#### 2.3.2 Probability analysis for robust evaluation

The workflow for the sNRV+R evaluation (1) and the conventional evaluation (2) is illustrated in Appendix C. Each considered dose metric *D*_*x*_ (e.g., *D*_95_) would have corresponding perturbed dose metrics under the different uncertainty scenarios. The nominal dose metric is subtracted from the perturbed dose metrics to form a distribution of dose metric discrepancies Δ$D_{x}$ experienced across the uncertainty scenarios.

The upper and lower boundaries of dose metrics in the evaluation can be demonstrated by the shaded areas in the nominal dose–volume histogram (DVH), as an indicator of worst‐case scenarios. It was also suggested in the literature to include a probability approach in robust analysis.^32^ For this, the distance between the probability distribution of Δ$D_{x}$ under uncertainty and its ideal probability distribution (Dirac delta function, the dose metrics do not change even under uncertainty) was calculated using the Wasserstein distance (WD)

(7) $$\mathrm{WD}\left(U,I\right)=\int_{-\infty }^{\infty }\left|U\left(x\right)-I\left(x\right)\right|dx,$$

where *U* and *I* are the probability distribution functions of Δ$D_{x}$ under uncertainty and its ideal distribution, respectively. The WD measures the effort required to convert one distribution into the other. The smaller the WD, the more robust is a plan for this dose metric.

#### 2.3.3 Performance analysis of robust evaluations

To investigate the effectiveness of sNRVs in indicating the plan robustness to inter‐fractional anatomical changes before treatment, the dose discrepancy between accumulated dose using weekly CTs and the nominal dose was taken as the gold standard. In the gold standard evaluation, the dose distributions of the IMPT plans with different beam arrangements were calculated on six weekly CTs of each test patient. Because the accumulated dose is generally used in treatment evaluation and related to prognostics, the weekly dose was accumulated in the reference frame of the planning CT using the DIR algorithm of Niftyreg, referred to as Accu${}_{\text{Nom}}$. In the weekly dose calculation, although the isocenter was determined using the information from the rigid registration, the setup error (both rigid setup and sNRV) still existed. Thus, the difference between Accu${}_{\text{Nom}}$ and the nominal plan, referred to as $\Delta D_{\text{st}}$, represents the influences from total random errors, including actual progression uncertainty and setup uncertainty (both rigid setup and sNRV).

Because different beam arrangements are used in this study, the robustness of beam arrangements can be ranked, referred to as robustness ranking. In the sNRV+R evaluation and the conventional evaluation, the WD is used in robustness ranking for each dose metric. In the gold standard evaluation, $\Delta D_{\text{st}}$ is used in robustness ranking. To quantitatively validate that the performance of the sNRV+R evaluation is better than the conventional evaluation, the consistency *C* of the robustness ranking for a dose metric is calculated for each beam arrangement of each patient, using

(8) $$C=|RP_{s}\left(B_{i}\right)-RP_{G}\left(B_{i}\right)|-|RP_{c}\left(B_{i}\right)-RP_{G}\left(B_{i}\right)|,$$

$RP_{s}\left(B_{i}\right)$, $RP_{c}\left(B_{i}\right)$, and $RP_{G}\left(B_{i}\right)$ represent the robustness ranking position of a beam arrangement $B_{i}$ in sNRV+R evaluation, conventional evaluation and the gold standard evaluation, respectively. If C≤0 for a dose metric, then this dose metric of beam arrangement $B_{i}$ supports that sNRV+R evaluation is better for robust evaluation, compared to the conventional rigid setup evaluation.

## 3 RESULT

### 3.1 Dosimetric influences caused by small non‐rigid variations

This section demonstrates the additional dosimetric influence caused by non‐rigid setup uncertainty.

**FIGURE 1:**
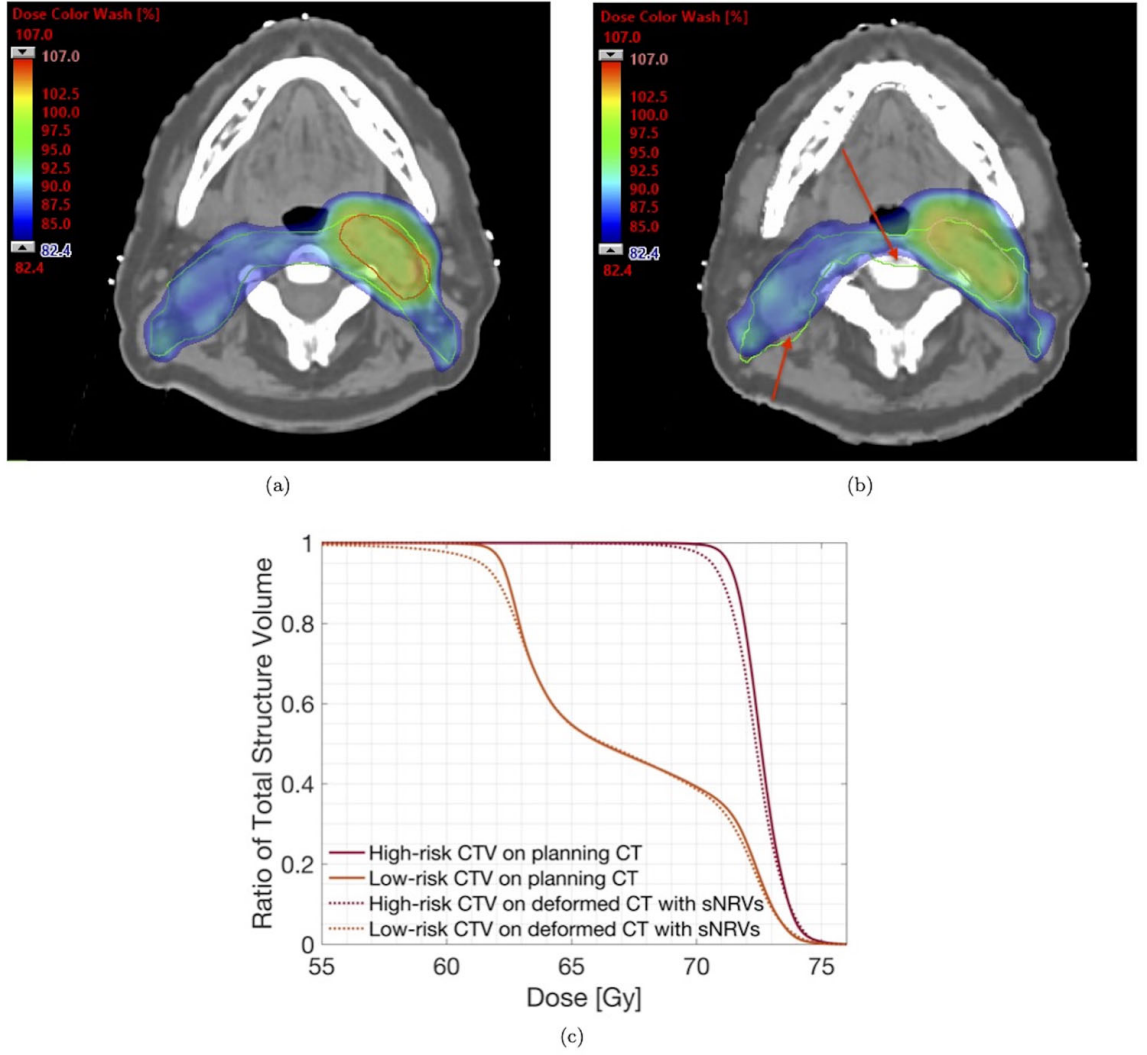
An example of dose distribution variations caused by a random small non‐rigid variation (sNRV). (a) The dose distribution on the planning CT, and (b) the dose distribution of the same slice on a deformed planning CT. The green contour in images is the low‐risk CTV. The color bar was chosen to mask out doses lower than the 95% prescription dose of low‐risk CTV (82.4% is corresponding to 95% prescription dose of low‐risk CTV). The red arrows indicate areas of underdosage caused by the sNRVs. (c) presents the difference in dose–volume histogram (DVH) caused by the sNRV

An example of the dose distribution difference caused by an sNRV is shown in Figure 1. The red arrows indicate areas where the dose has fallen under 95% of the prescription dose. With these simulated images of sNRVs and corresponding dose distributions, we can help clinicians to avoid non‐rigid postures that can lead to unacceptable dosimetry.

**FIGURE 2:**
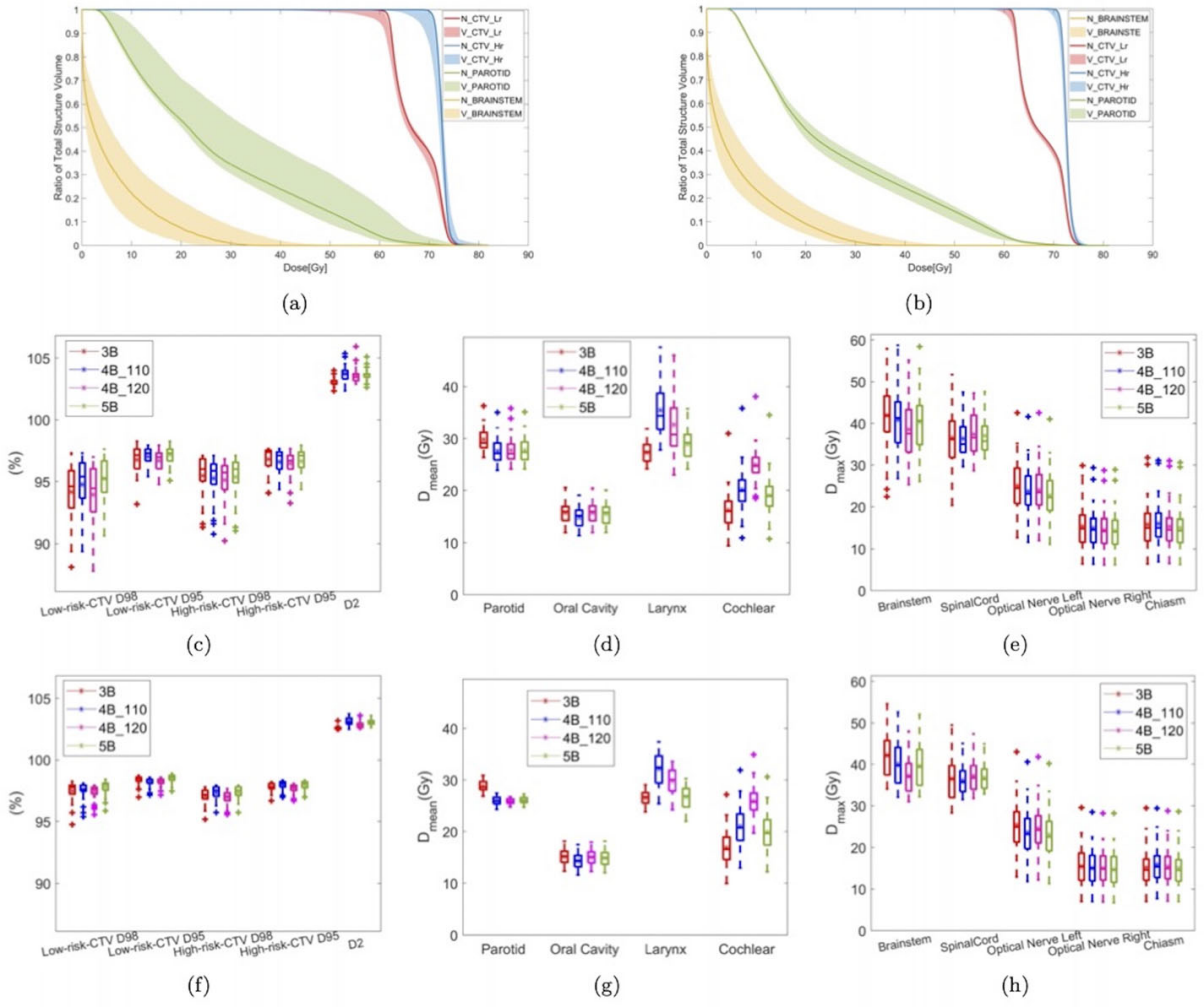
The comparison between the sNRV+R evaluation and the conventional evaluation on patient 1. (a,b) The shaded DVH from the 4*B*_120_ beam arrangement in the sNRV+R evaluation and in the conventional evaluation, respectively. The solid line represents the DVH of the nominal plan (N in the legend), the shaded area indicates the lower and upper boundary of dose metrics in the respective evaluation, caused by the variations (V in the legend). (c–h) Visually summarize the statistics under the respective uncertainty using box plots. The horizontal lines in the box plot indicate the median dose metric among 36 scenarios (including the nominal scenario and 35 uncertainty scenarios defined in robust evaluation). The bottom and top edges of the box indicate the 25th and 75th percentiles, respectively. We use the asterisks to indicate the mean value of the dose metrics. (c–e) The boxplots of *D*_*x*_ in the sNRV+R evaluation. (f–h) are the boxplots of *D*_*x*_ in the conventional evaluation

The comparison between the sNRV+R evaluation and the conventional evaluation on an exemplary patient (patient 1) is shown in Figure 2. The upper and lower boundaries of dose metrics in the sNRV+R evaluation (2a) and the conventional evaluation (2b) are indicated by the shaded areas in Figure 2(a,b) separately. We observed that the additional sNRVs widen the bandwidth compared to the conventional robust evaluation. The detailed numbers of the sNRV+R evaluation and the conventional evaluation for four test patients are listed in Appendix D. Among four patients (490 scenarios), we observed a maximum difference in the sNRV+R evaluation to the nominal dose of: 9.37% dose degradation on the *D*_95_ of CTVs, increase in parotid *D* ${}_{\text{mean}}$ by 11.87 Gy, increase in larynx *D* ${}_{\text{mean}}$ by 15.04 Gy, increase in brainstem *D* ${}_{\max}$ by 20.82 Gy, increase in spinal cord *D* ${}_{\max}$ by 20.96 Gy. For CTVs, 4 patients all had scenarios where the CTV D_95_ fell below 95%, 47 out of 490 scenarios in total. In contrast, in conventional evaluation, we observed a maximum difference to the nominal dose of: 7.58% dose degradation on *D*_95_ of the CTVs, increase in parotid *D* ${}_{\text{mean}}$ by 4.88 Gy, increase in larynx *D* ${}_{\text{mean}}$ by 6.13 Gy, increase in brainstem *D* ${}_{\max}$ by 13.5 Gy, and increase in spinal cord *D* ${}_{\max}$ by 12.9 Gy.

Please note that the worst‐case CTV coverage (*D*_95_) under setup uncertainty can drop below 95% in some cases. To generate 35 scenarios in conventional robust evaluation, we let the isocenter shifts follow a Gaussian distribution with mean μ= 0 mm and standard deviation σ= 1.5 mm. This results in multiple scenarios that can be used for statistical analysis, rather than only using the 12 scenarios usually encountered during robust optimization with 3 mm orthogonal shifts and ±3.5% range error. While we still used the usual 3 mm option to optimize the plan, the additional shifts created with the Gaussian distribution were used for the evaluation. Using this Gaussian distribution may result in scenarios where the shift exceeds 3 mm. However, only 4/490 scenarios were below 95%. Those scenarios only happened to patient 3 whose target volume was located close to the skin, making this particular patient more sensitive to setup uncertainties.

The comparisons of the dose metrics for this patient based on box plots are shown in Figure 2(c)–(h). Dose metrics for the different plans with different beam arrangements are shown in the same figures as box plots. By comparing boxplot of (c)–(e) (sNRV+R evaluation) to (f)–(h) (conventional evaluation) in Figure 2, the mean values of the CTVs' D_95_ in the sNRV+R evaluation are lower than the values in the conventional evaluation ranging from −1.57% to −0.95% (range shows the differences between different beam arrangements). The mean values of parotid *D*_mean_, oral cavity *D*_mean_, and larynx *D*_mean_ are higher than the values in conventional evaluation, ranging from 1.02 to 1.82 Gy, 0.52 to 0.70 Gy, and 0.84 to 3.18 Gy, respectively. The mean values of *D* ${}_{\max}$ of spinal cord, optical nerve, and chiasm between the two evaluations only have slight differences, less than 0.6 Gy.

**FIGURE 3:**
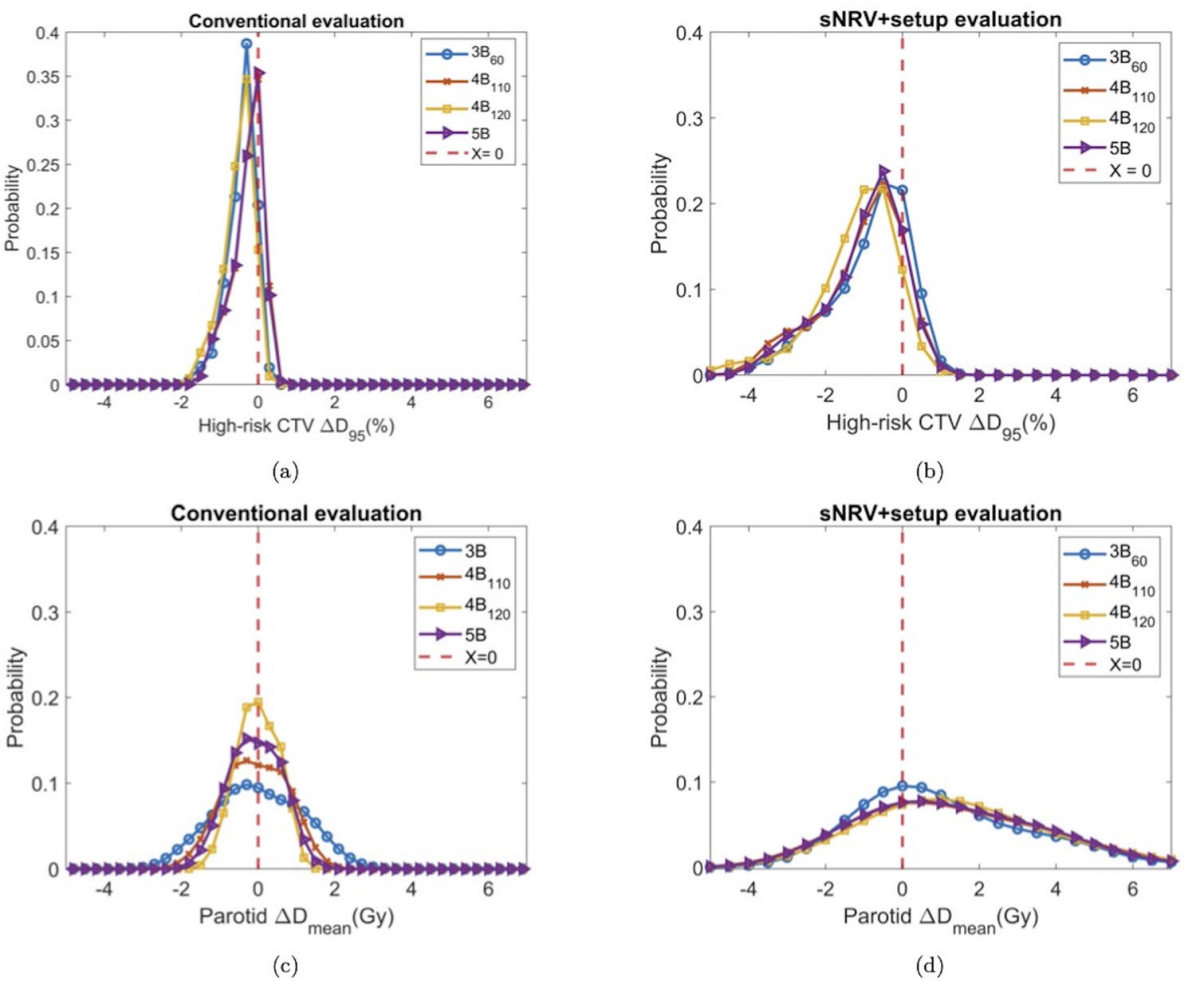
Probability distributions of Δ*D*_*x*_ in the conventional evaluation and in the sNRV+R evaluation on patient 1. (a) Probability distribution of Δ*D*_*x*_ in the conventional evaluation on the high‐risk CTV D_95_. (b) Probability distribution of Δ*D*_*x*_ in the sNRV+R evaluation on the high‐risk CTV *D*_95_.(c) Probability distribution of Δ*D*_*x*_ in the conventional evaluation on the parotid *D* ${}_{\text{mean}}$. (d) Probability distribution of Δ*D*_*x*_ in the sNRV+R evaluation on the parotid *D* ${}_{\text{mean}}$

Figure 2 only partially demonstrates the Dx under uncertainty. In Figure 3, we plot the probability distribution of ΔDx in the conventional evaluation and in the sNRV+R evaluation on high‐risk CTV D_95_ and parotid D${}_{\text{mean}}$, respectively, for patient 1. In Figure 3, we can see the influence caused by the sNRVs on the probability distribution of ΔDx from different beam arrangements. The robustness of a beam arrangement is presented by the closeness of the probability curve of beam arrangements to the Dirac delta function. For the high‐risk CTV, the 3B_60_ plan is the most robust (the Δ*D* ${}_{\text{mean}}$ curve of the 3*B*_60_ is the closest to the Dirac delta function, indicated as the dashed vertical line) in the sNRV+R evaluation, as opposed to the conventional evaluation, where we find this beam arrangement to be the less robust one. For the parotid glands, in conventional evaluation (Figure 3c)), the 4*B*_120_ is the most robust beam arrangement, while in sNRV+R evaluation (Figure 3d)), the 3*B*_60_ is the most robust. The $\Delta D_{st}$ from the gold standard evaluation validated that 3*B*_60_ indeed is the most robust beam arrangement for the parotid *D* ${}_{\text{mean}}$ (please refer to the table in Appendix D).

### 3.2 Robust evaluation analysis

This section applies the ranking consistency to demonstrate the benefit of sNRVs for robust evaluation.

Regarding the 10 robustly optimized dose metrics listed in the lower part of Table 1 for each beam arrangement, we calculated the percentage of dose metrics that supports the inclusion of sNRV in robust evaluation based on the data of four test patients. Referring to the table in Appendix D, we summarized the percentage of dose metrics satisfying C≤0 (*P* ${}_{C\leq 0}$) for each beam arrangement in Table 2. *P* ${}_{C\leq 0}$s are all above 70%, showing that overall including the sNRVs is beneficial to the robust evaluation of all beam arrangements.

**TABLE 2:** Consistency of robustness ranking between two robust evaluation methods for each beam arrangement

Beam arrangements	3B45	3B60	4B110	4B120	5B
***P*_C≤0_(%)**	90	77.5	70	75	72.5

*Note*: The *P*_C≤0_ summarizes the percentage of the ROI metrics that supports the sNRV+R evaluation (*C* ≤ 0) for each beam arrangement.

We summarized the percentage of C≤0 across all patients and beam arrangements for each dose metric in Table 3. Here, we can conclude that overall including the sNRVs is beneficial to robust evaluation for each dose metric, compared to only include rigid setup uncertainty.

**TABLE 3:** Consistency of robustness ranking between two robust evaluation methods for each robustly optimized dose metric

**Dose metric**	**High‐risk CTV**	**Low‐risk CTV**	**Parotid**	**Cochlea**	**Brainstem**	**Spinal cord**	**Chiasm**	**Optical nerve**
	***D*_95_**	***D*_95_**	***D*mean**	***D*mean**	***D*max**	***D*max**	***D*max**	***D*max**
	**(%)**	**(%)**	**(Gy)**	**(Gy)**	**(Gy)**	**(Gy)**	**(Gy)**	**(Gy)**
*P_C_*_≤0_ (%)	64.29	78.57	85.71	85.71	64.29	92.86	78.57	71.43

*Note*: The *P_C_*_≤0_ summarizes the percentage of *C*≤0 over all patients and beam arrangements for each dose metric.

## 4 DISCUSSION

Dose distributions in proton therapy are more sensitive to geometric changes than photon therapy. However, in previously published methods of robust evaluation, the impact of anatomical changes before treatment was not considered. In this paper, we demonstrated that including sNRVs into robust evaluation is beneficial.

### 4.1 The use of small non‐rigid variations for robust beam selection

In the validation of sNRVs' role in robust evaluation, the dose discrepancy that represents the influence from inter‐fractional anatomical changes and isocenter shifts was used as the gold standard. The consistency of robustness ranking showed that *P* ${}_{C\leq 0}$ is higher especially on the spinal cord, parotid gland and low‐risk CTV, which are closely related to outline changes and neck motions, and also on small structures really sensitive to the sNRVs such as cochlea and chiasm, supporting that sNRVs play a positive role in robust evaluation in terms of indicating robustness to inter‐fractional anatomical changes.

The method proposed in this study can assist in selecting robust beam arrangements for proton plans without 4D optimization. In Appendix D, the *p*‐values between the distributions of Δ$D_{x}$ in the sNRV+R evaluation and conventional evaluation showed that the sNRVs mainly influenced the probability distribution of CTVs Δ*D*_95_ and parotid Δ*D* ${}_{\text{mean}}$. The highest priority of the robust optimization for the four test patients in this study was to ensure target coverage. Similar performance of *D*_95%_ based on $\Delta D_{\text{st}}$ was found on different beam arrangements, with differences smaller than 2%. To best demonstrate the advantage of the sNRV+R evaluation over the conventional evaluation, the beam arrangement was selected based on the impact of the sNRVs on the dose of parotid glands as an illustration. Also, the dose on parotid glands is closely related to toxicity such as xerostomia and dysphagia that can have a long‐term impact on patients' quality of life. Here, for example, for patient 1, a similar parotid *D* ${}_{\text{mean}}$ was achieved using 4*B*_110_ and 4*B*_120_. If 4*B*_120_ was selected based on WD, the accumulated parotid *D* ${}_{\text{mean}}$ reduces by 0.7 Gy, which is corresponding to 1 fraction of *D* ${}_{\text{mean}}$ delivered to the parotid glands. Other organs can be used for beam selection as well, for example, for patient 1, the rank of the chiasm *D* ${}_{\max}$ in the sNRV+R evaluation indicated the most robust beam arrangement as the gold standard evaluation.

There were two interesting scenarios worth noticing. In different beam arrangements for patient 1, even though the nominal parotid *D* ${}_{\text{mean}}$ of 3*B*_60_ was the highest, the accumulated dose was lower than 4*B*_110_ and 5*B* because 3*B*_60_ was the most robust beam arrangement (the lowest WD) under sNRV+R uncertainty. The $\Delta D_{\text{st}}$ of 3*B*_60_ showed that 3*B*_60_ controls $\Delta D_{\text{st}}$ of the parotid *D* ${}_{\text{mean}}$ within 3 Gy, which is corresponding to 10% NTCP difference^33^ and used to trigger replan to protect the parotid glands. This case indicates that beam selection based on robust evaluation can potentially reduce the replan rate, something that needs further investigation in the future. For patient 3, even though the nominal parotid *D* ${}_{\text{mean}}$ of 4*B*_120_ was higher than in the 5*B* beam arrangements, the accumulated dose was the lowest because 4*B*_120_ was the more robust beam arrangement. A message clearly emerged here is that the best nominal plan may not be the best plan during the treatment.

The impact of different beam angles on the robustness of a plan can be analyzed on the patient‐specific geometry using our method. The results can be used to create a robustness plan database to assist to find a more robust planning approach as presented by McGowan et al.^22^ and Malyapa et al.^23^

### 4.2 The potential use of small non‐rigid variations in clinic

The distribution of sNRVs has the potential to be used in other clinical applications.

First, we found that the sNRV that leads to the most dose discrepancy varies from patient to patient and from beam arrangement to beam arrangement. This approach can help clinicians avoid the set‐ups with the sNRVs that can lead to unacceptable dose distributions for a specific patient using a specific beam arrangement.

Second, the acquired sNRVs can potentially assist in better estimating the truly delivered accumulated dose using weekly CTs. The sNRVs can be randomly allocated to each weekly CT, with 5 sNRVs per weekly CT. These deformed weekly CTs can be used to estimate the daily dose distribution under the influence of sNRV. Repeating this procedure can reveal the range of potential accumulated doses for the whole treatment.

Third, this study presented the possibility of including sNRVs from a patient population to robust analysis, which also indicated the potential to be used in robust optimization. Mesías et al.^15^ included the first two weekly CTs of patients into robust optimization to account for the sNRVs, suggesting that sNRVs can reduce the need for adaption. They indicated that the first two weekly CTs can be replaced by a series of CT images scanned before treatment. Li et al.^34^ considered weekly CTs in the robust evaluation, and Yang et al.^35^ added the adaptive planning CTs into robust optimization. However, their methods relied on the acquisition of CT images during the treatment, which limits the creation of a robust plan at the early planning stage. In contrast to their patient‐specific approach, I suggest an atlas‐based technique. While this approach is not patient‐specific but based on the assumption that sNRVs are mainly random, there are some advantages. First, this method does not require the acquisition of a series of CT images of the same patient pre‐treatment, therefore saving imaging dose and reducing workload. Second, assuming that sNRVs can be reasonably represented using this method, deformed images with the sNRVs can be prepared in advance and fully exploit the benefits of robust optimization with multiple CTs. This will be investigated in future studies.

It should be mentioned that the inclusion of a large patient cohort (many sNRV scenarios) would require recalculating the treatment plan many times. For efficiency, I suggest limiting the number of included sNRVs to the most common/frequent ones. The most common sNRVs can be found, for example, by using anatomical models which use principal component analysis applied to anatomical deformations of a patient cohort to estimate the likelihood of a certain anatomical deformation to happen. By only including the most likely principal components of the deformation into the robust evaluation, the number of recalculated plans can be reduced while still representing well the sNRVs. This trade‐off will be explored in future work.

The concept presented here can be adapted to different scenarios. Here, I did not factor in immobilization equipment and patient characteristics such as age, size, disease staging, and physical condition. All those factors are likely to influence the possibility and the amplitude of a specific anatomical change to arise during the treatment. While this is not yet considered in this paper, the presented approach has the potential to do so. If sufficient patient data are used to build the atlas, the patient data can be stratified into groups of different immobilization devices and patient characteristics before performing the robust evaluation.

### 4.3 Limitations

For the purpose of showing the feasibility, different plans with different beam arrangements were only created for four test patients. Further validation of the methods will be conducted on a large number of patients. Another limitation of this work is that the impact of DIR accuracy was ignored on robust evaluation. We assumed that the DIR uncertainty would equally affect the robust evaluation for different beam arrangements.

When validating the role of sNRVs in robust evaluation, we decided to not take the range uncertainty from Hounsfield units (HU) into account because range uncertainty is an isolated source considered in the robust evaluation and is solely based on the CT calibration. Therefore, it should only have small influence on the results of the comparison, which established that sNRV should be considered in the robust evaluation as a component of the random set up errors, not just rigid set up. However, to fully evaluate the plan, the range uncertainty should be used with the translation rigid setup and sNRVs. Please see Appendix E for an example of the further evaluation.

## 5 CONCLUSION

This study demonstrated the additional dose discrepancy arising from sNRVs and the robust plan evaluation method based on the probability distribution. The novelty of this study exists in three aspects: (1) compared with the conventional evaluation, we demonstrated that the inclusion of sNRVs can be beneficial to robust evaluation in terms of indicating plan robustness to inter‐fractional anatomical changes. (2) This atlas‐based method can help clinicians to choose plans that are robust against those sNRVs. (3) Our method can potentially provide multiple images for potential future 4D optimization.

## CONFLICT OF INTEREST

The authors declare that there is no conflict of interest that could be perceived as prejudicing the impartiality of the research reported.

## Supporting information

Supporting Information

## Data Availability

Restricted access. The authors cannot make these data publicly available due to data use agreement.

## Footnotes

1
https://cmiclab.cs.ucl.ac.uk/mmodat/niftyreg
