# Supplementary material for: Pre‐treatment analysis of non‐rigid variations can assist robust intensity‐modulated proton therapy plan selection for head and neck patients
Source: Med Phys. 2022 Oct 6;49(12):7683–93. doi: 10.1002/mp.15971 (PMC10092578; doi:10.1002/mp.15971)
Supplement: Supplementary file 1 — Supporting Information [file MP-49-7683-s001.pdf]

Appendix A: Examples of small non-rigid variations.

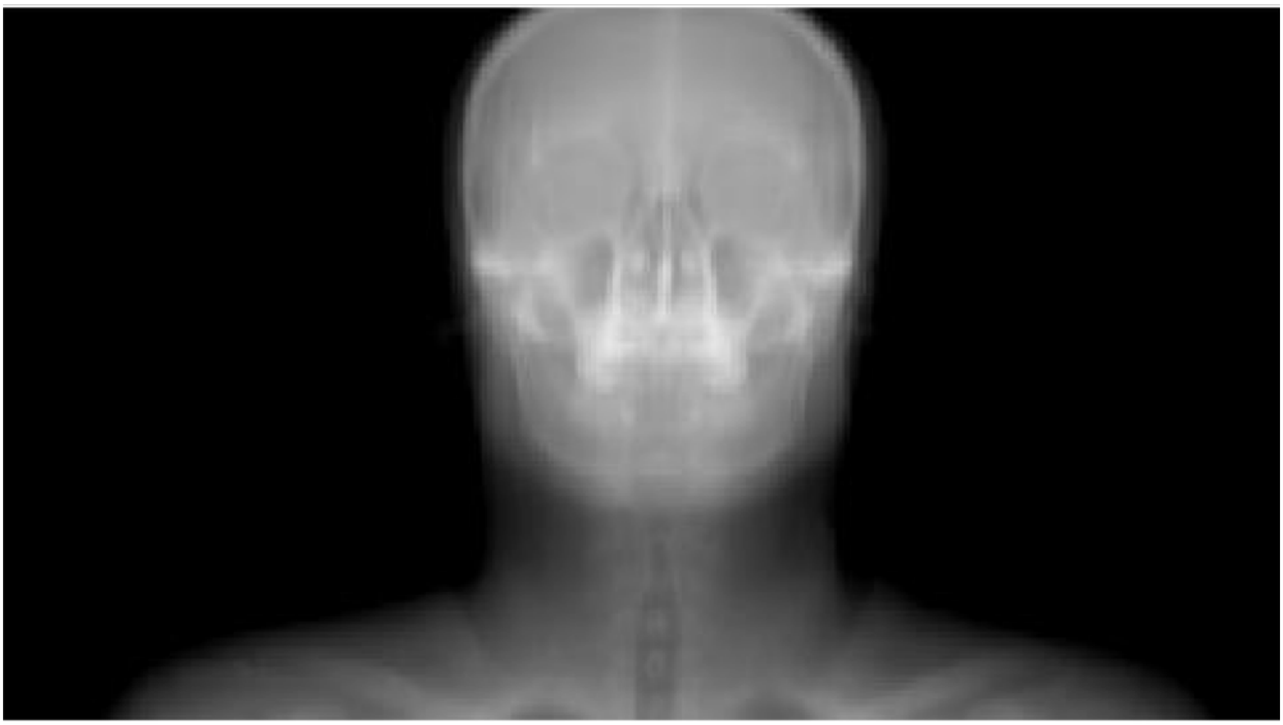

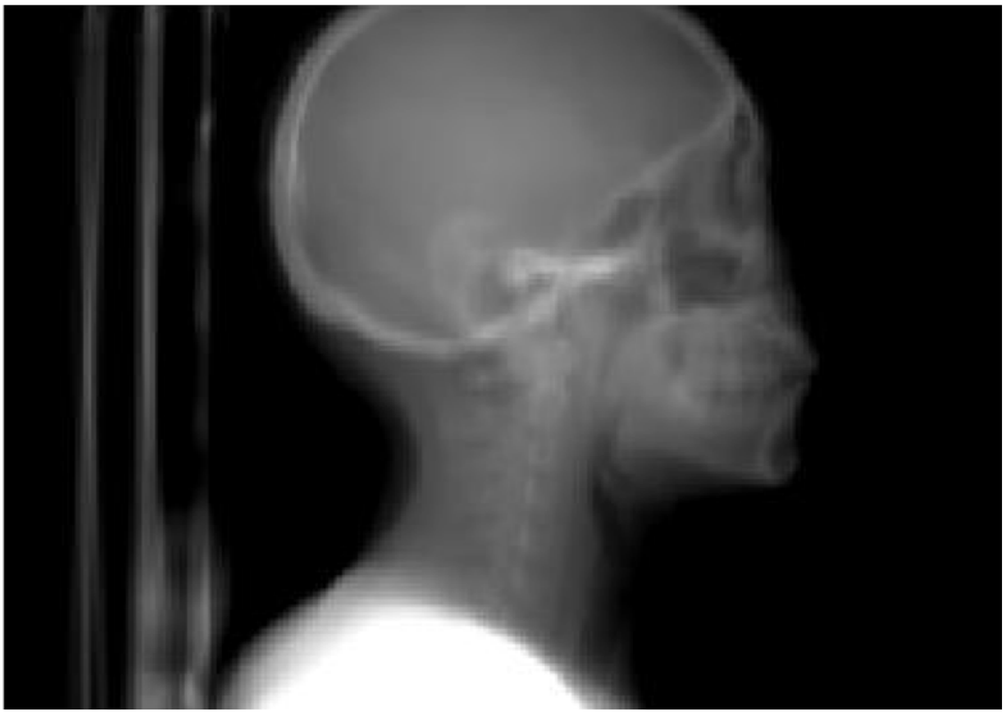

Appendix B: Beam arrangements illustration.

For our patients, experienced clinicians choose beam angles based on individual patients' geometry. Therefore, beam arrangements are not exactly the same for each patient. Therefore, beam arrangements are not exactly the same for each patient.

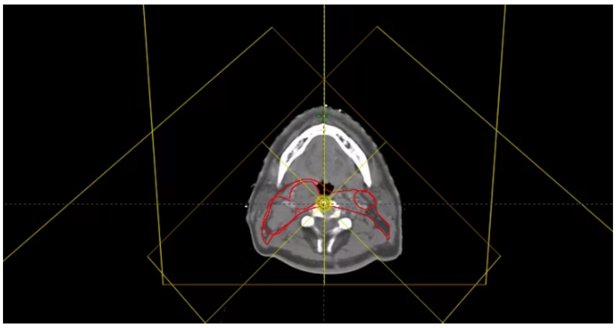

(a)

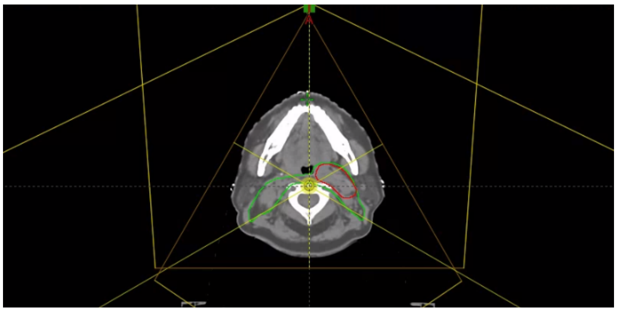

(b)

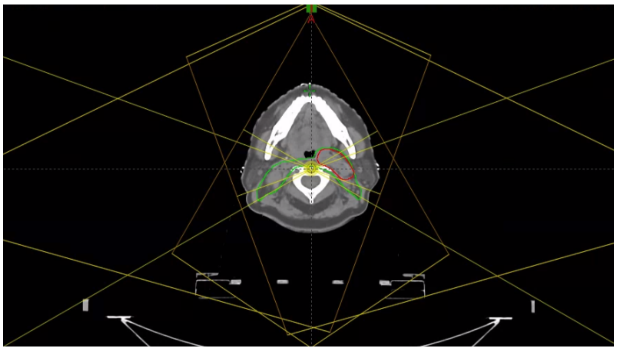

(c)

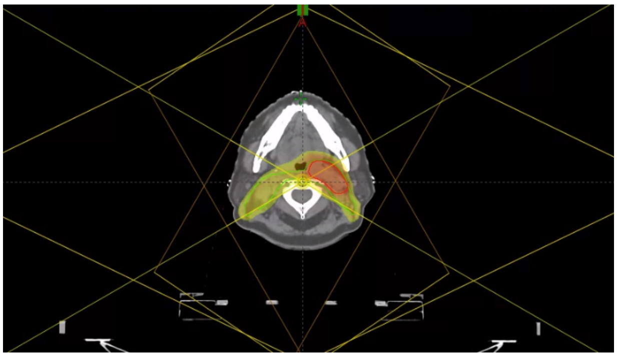

(d)

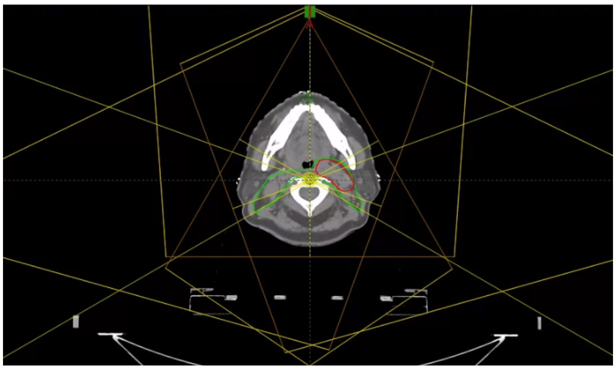

(e)

FIG. 1: Illustration of different beam arrangements. (a) - (e) are $3B_{45}$, $3B_{60}$, $4B_{110}$, $4B_{120}$, 5B, respectively.

Appendix C: Workflow

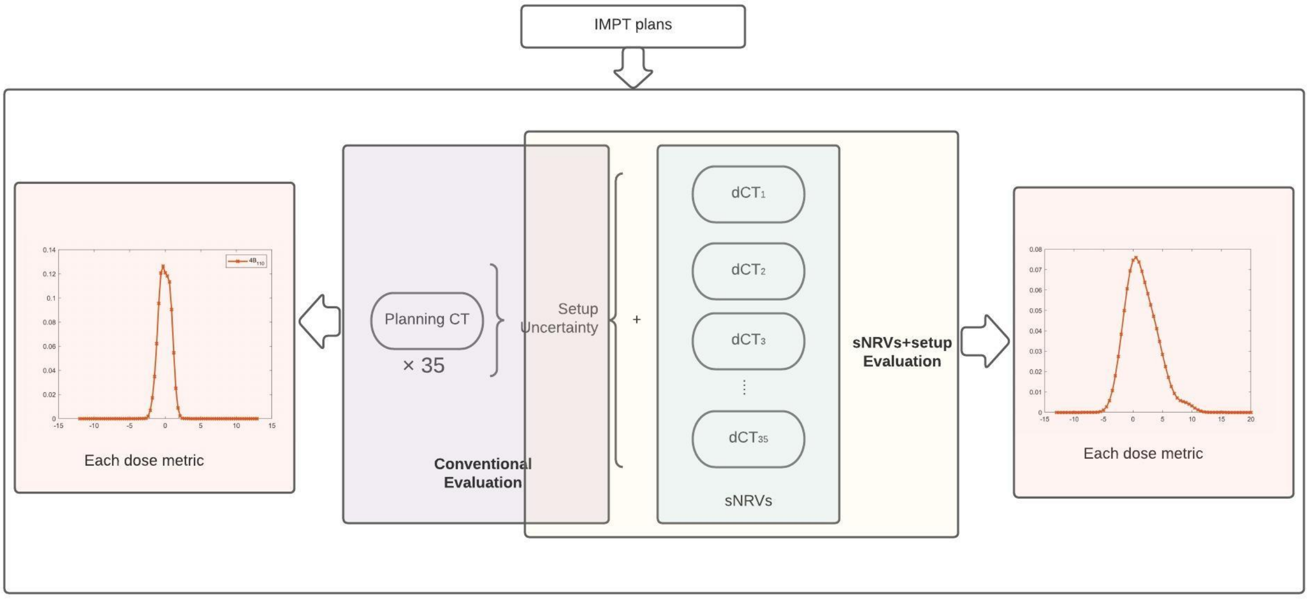

FIG. 2: The workflow of sNRV+R evaluation and conventional evaluation for each IMPT plan. Both evaluation methods produce 35 perturbed dose distributions. Each considered dose metric (Dx, e.g. D_{95}) would have corresponding perturbed dose metrics under the different uncertainty scenarios. The nominal dose metric is subtracted from the perturbed dose metrics to form a distribution of dose metric discrepancies (ΔDx) experienced across the uncertainty scenarios. The light yellow box indicates that IMPT plans were calculated on uncertainty scenarios from sNRVs plus setup in the sNRV+R evaluation. The light purple box indicates that IMPT plans were calculated on setup uncertainty scenarios in the conventional evaluation. The dose distribution of ΔD_{95} in the conventional evaluation and the sNRV+R evaluation was plotted in the left organ box and the right organ box, respectively, as an illustration.

Appendix D: Robust evaluation table for four test patients.

TABLE D1: The sNRV+R evaluation, conventional evaluation and gold standard evaluation for four test patients. p-values of the two-sample t-test are calculated between the distribution of ΔDx in the sNRV+R evaluation and in the conventional evaluation. p<0.05 represents ΔDx in the sNRV+R evaluation and ΔDx in the conventional evaluation are taken from significantly different distributions. In the sNRV+R evaluation and the conventional evaluation, the beam arrangements were ranked based on the WD for each ROI matrix. In the gold standard evaluation, the beam arrangements were ranked for each ROI matrix based on ΔD_{st}. RP is the robustness ranking position of a beam arrangement for a matrix.

	sNRV+R Evaluation						Conventional Evaluation					Gold standard evaluation		
ROI/Matrix	Plan	Nominal	Min	Max	WD	RP_s	Minmum	Maximum	WD	RP_c	p-value	$Accu_{Nom}$	ΔD_{st}	RP_G
patient 1														
LOW-RISK CTV D95(%)	$3B_{60}$	98.57	93.17	98.25	1.68	4	96.83	98.57	0.37	3	1e-10	97.62	-0.95	3
	$4B_{110}$	98.25	95.4	97.94	1.22	1	96.83	98.25	0.32	2	1e-9	97.62	-0.63	2
	$4B_{120}$	98.25	94.76	97.94	1.57	3	96.83	98.25	0.29	1	1e-11	97.3	-0.95	4
	5B	98.57	95.08	98.25	1.4	2	97.14	98.57	0.39	4	1e-10	97.94	-0.63	1
HIGH-RISK CTV D95(%)	$3B_{60}$	97.66	94.08	97.66	0.83	1	96.14	97.66	0.46	3	0.01	97.66	0	1
	$4B_{110}$	97.66	94.35	97.66	1.08	4	96.42	97.8	0.28	1	1e-5	97.38	-0.28	3
	$4B_{120}$	97.66	93.25	97.66	1.05	3	96.14	97.52	0.52	4	1e-4	97.38	-0.28	3
	5B	97.66	94.35	97.93	1.04	2	96.42	97.8	0.32	2	1e-5	97.38	-0.28	3
HIGH-RISK CTV D2(%)	$3B_{60}$	102.89	102.34	103.99	0.18	1	102.75	103.58	0.11	1	0.9	101.79	-1.1	4
	$4B_{110}$	103.44	102.34	105.37	0.45	3	103.03	104.27	0.25	4	0.64	103.17	-0.28	2
	$4B_{120}$	102.89	102.89	105.92	0.66	4	102.75	103.86	0.17	2	1e-6	102.89	0	1
	5B	103.44	102.62	105.1	0.28	2	103.17	104.13	0.18	3	0.93	102.89	-0.55	3
PAROTID D_{mean}(Gy)	$3B_{60}$	28.62	26.36	36.31	1.68	1	26.68	30.74	0.77	4	1e-3	31.48	2.86	1
	$4B_{110}$	26.11	23.89	35.07	2.04	4	24.41	27.51	0.55	3	1e-3	31.82	5.72	3
	$4B_{120}$	25.94	24.14	35.81	1.97	3	24.85	26.77	0.34	1	1e-4	31.1	5.16	2
	5B	26.29	24.15	35.14	1.8	2	24.88	27.42	0.43	2	1e-4	32.24	5.95	4
ORAL D_{mean}(Gy)	$3B_{60}$	15.17	11.98	20.57	1.6	2	12.42	18.17	1.14	3	0.24	15.56	0.38	2
	$4B_{110}$	14.33	11.38	19.06	1.56	1	11.69	17.53	1.13	2	0.26	14.7	0.37	1
	$4B_{120}$	15.05	11.99	20.46	1.63	4	12.36	18.01	1.08	1	0.13	15.71	0.66	4
	5B	14.87	11.99	20.07	1.62	3	12.1	18.18	1.16	4	0.23	15.27	0.39	3
LARYNX D_{mean}(Gy)	$3B_{60}$	26.6	24.18	31.89	1.71	1	24.09	29.39	1.05	1	0.17	28.96	2.36	1
	$4B_{110}$	32.21	27.81	47.64	4.22	3	25.66	37.63	2.55	4	1e-3	42.42	10.2	3
	$4B_{120}$	30.11	22.99	46.12	4.27	4	24.64	34.01	1.97	3	0.02	44.4	14.29	4
	5B	26.78	24.11	35.75	2.76	2	22.31	30.6	1.72	2	1e-3	33	6.22	2
COCHLEA D_{mean}(Gy)	$3B_{60}$	15.53	9.45	30.97	2.38	2	9.09	26.29	2.65	2	0.83	13.79	-1.74	4
	$4B_{110}$	20.21	10.95	35.83	2.53	4	12.53	31.56	3.01	4	0.63	18.51	-1.69	3
	$4B_{120}$	25.11	18.49	38.08	1.89	1	19.38	34.61	2.24	1	0.41	23.43	-1.68	2
	5B	19.02	10.75	34.52	2.5	3	11.72	30.11	2.88	3	0.65	17.46	-1.56	1
BRAINSTEM D_{max}(Gy)	$3B_{60}$	43.02	22.42	57.93	5.83	3	34.72	55.22	4.2	4	0.57	40.84	-2.18	4
	$4B_{110}$	40.26	26.75	58.72	5.98	4	32.46	52.96	4.18	3	0.75	40.54	0.29	2
	$4B_{120}$	36.43	25.33	55.17	5.57	1	30.73	47.73	3.39	1	0.25	37.63	1.2	3
	5B	39.51	26.06	58.4	5.79	2	32.41	52.31	4.07	2	0.71	39.71	0.21	1
SPINAL D_{max}(Gy)	$3B_{60}$	36.68	20.36	51.68	5.6	4	28.38	49.58	4.22	4	0.96	33.25	-3.43	4
	$4B_{110}$	35.11	29.63	47.5	3.54	2	30.81	44.31	2.57	2	0.34	34.92	-0.2	1
	$4B_{120}$	36.65	28.64	47.28	4.08	3	31.85	47.35	2.97	3	0.95	34.59	-2.07	3
	5B	35.47	31.55	47.61	3.11	1	31.87	43.87	2.31	1	0.13	36.56	1.09	2
CHIASM D_{max}(Gy)	$3B_{60}$	23.68	12.7	42.56	4.88	4	12.28	42.38	4.78	4	0.9	22.79	-0.89	3
	$4B_{110}$	22.29	11.61	41.64	4.52	2	11.29	40.09	4.57	1	0.67	21.41	-0.88	2
	$4B_{120}$	22.9	12	42.53	4.62	3	11.7	41.3	4.63	3	0.96	21.9	-0.99	4
	5B	21.62	11.11	41.03	4.51	1	10.72	39.62	4.61	2	0.88	20.77	-0.85	1
OPTIC_NERVE_L D_{max}(Gy)	$3B_{60}$	13.97	6.37	29.86	4.05	4	6.27	28.87	3.72	4	0.56	16.44	2.47	4
	$4B_{110}$	13.6	6.32	29.38	3.75	3	6.2	27.7	3.49	3	0.52	15.3	1.7	1
	$4B_{120}$	13.44	6.15	28.74	3.65	2	6.14	27.44	3.47	1	0.79	15.64	2.19	3
	5B	13.23	6.18	28.9	3.65	1	5.83	27.33	3.48	2	0.61	15.16	1.93	2
OPTIC_NERVEV_R D_{max}(Gy)	$3B_{60}$	13.97	6.37	31.77	4.22	4	6.37	28.87	3.54	4	0.3	19.74	5.77	4
	$4B_{110}$	14.37	6.92	23.76	3.14	3	6.77	28.47	3.47	3	0.33	19.81	5.44	1
	$4B_{120}$	13.75	6.5	23.25	3.09	2	6.15	27.85	3.4	1	0.44	19.31	5.56	2
	5B	13.42	6.24	22.81	3.06	1	6.02	27.62	3.43	2	0.4	18.99	5.57	3

TABLE D1: Continued

ROI/Matrix	Plan	sNRV+R Evaluation Nominal	Min	Max	WD	RP	Conventional Evaluation Minmum	Maximum	WD	RP	p-value	Gold standard evaluation Accu_{Nom}	ΔD_{st}	RP
patient 2														
Low-risk-CTV D95(%)	$3B_{45}$	98.25	92.22	97.94	1.6	3	96.67	98.25	0.36	4	1e-8	97.94	-0.32	3
	$3B_{60}$	98.25	93.17	98.25	1.92	4	96.03	98.25	0.3	2	1e-9	97.94	-0.32	3
	$4B_{120}$	98.25	93.81	98.25	0.97	1	95.71	98.25	0.36	3	1e-5	97.94	-0.32	3
	5B	98.57	94.44	98.57	1.57	2	96.98	98.57	0.26	1	1e-8	98.57	0	1
CTV-T D95(%)	$3B_{45}$	98.48	95.73	98.48	0.87	2	96.56	98.48	0.4	2	1e-4	98.21	-0.28	2
	$3B_{60}$	98.48	93.8	98.21	1.03	4	96.56	98.48	0.42	3	1e-6	97.93	-0.55	4
	$4B_{120}$	98.48	94.9	98.21	0.89	3	96.56	98.48	0.47	4	1e-5	98.21	-0.28	2
	5B	98.48	95.45	98.48	0.77	1	96.56	98.48	0.34	1	1e-4	98.21	-0.28	2
CTV-T D2(Gy)	$3B_{45}$	102.34	102.07	103.17	0.23	2	101.93	102.89	0.11	3	0.11	101.79	-0.55	2
	$3B_{60}$	102.62	102.34	103.17	0.12	1	102.48	103.03	0.03	1	0.93	102.07	-0.55	3
	$4B_{120}$	102.34	102.07	103.99	0.5	4	102.07	103.44	0.23	4	0.02	103.17	0.83	4
	5B	102.07	101.52	103.17	0.31	3	101.93	102.2	0.04	2	1e-5	102.34	0.28	1
PAROTID D_{mean}(Gy)	$3B_{45}$	21.69	19.13	29.05	1.22	1	18.72	26.57	1.05	4	0.01	24.67	2.98	1
	$3B_{60}$	24.96	22.79	31.33	1.26	2	22.48	28.43	0.76	2	1e-3	28.2	3.24	2
	$4B_{120}$	23.07	20.8	31.53	1.86	3	20.97	24.98	0.77	3	1e-6	27.51	4.44	3
	5B	23.47	20.52	31.15	1.9	4	21.78	26.7	0.57	1	1e-4	28.6	5.12	4
ORAL D_{mean}(Gy)	$3B_{45}$	27.36	22.78	31.92	1.64	4	24.79	31.63	0.88	1	0.24	27.76	0.41	4
	$3B_{60}$	12.15	9.46	16.01	1.37	2	9.59	15.67	0.93	2	0.22	12.13	-0.01	1
	$4B_{120}$	11.33	8.74	15.55	1.36	1	7.46	14.38	0.98	4	0.04	11.44	0.11	3
	5B	12.13	9.41	15.66	1.38	3	9.5	15.63	0.95	3	0.11	12.2	0.07	2
LARYNX D_{mean}(Gy)	$3B_{45}$	16.7	13.14	23.63	1.71	1	15.22	19.48	0.83	1	0.2	19.81	3.11	2
	$3B_{60}$	17.13	13.61	23.81	1.76	2	15.18	20.12	0.9	2	0.11	19.6	2.47	1
	$4B_{120}$	16.62	11.21	32.2	3.44	4	9.32	23.07	2.13	4	0.01	25.44	8.82	4
	5B	17.76	14.44	26.44	2.42	3	15.53	21.5	0.98	3	0.01	21.17	3.41	3
COCHLEAR D_{mean}(Gy)	$3B_{45}$	19.01	14.96	25.91	2.29	3	15.59	24.35	1.75	1	0.24	26.63	7.62	4
	$3B_{60}$	13.33	8.24	25.87	2.39	4	7.72	20.88	2.27	4	0.45	13.75	0.42	3
	$4B_{120}$	21.67	18.09	29.09	1.87	1	17.98	32.11	1.99	2	0.73	21.31	-0.36	2
	5B	20.7	16.57	29.46	2.26	2	15.36	27.77	2.09	3	0.65	20.52	-0.18	1
BRAINSTEM D_{max}(Gy)	$3B_{45}$	32.23	27.74	65.33	9.45	4	24.03	43.13	3.26	1	1e-6	35.4	3.16	4
	$3B_{60}$	39.17	30.07	59.99	5.37	2	26.57	50.67	3.95	3	0.11	38.91	-0.26	1
	$4B_{120}$	41.35	28.71	60.47	5.38	3	29.15	57.95	4.49	4	0.91	38.86	-2.49	3
	5B	43.21	31.31	63.29	4.11	1	30.91	53.71	3.79	2	0.9	41.77	-1.44	2
SPINAL D_{max}(Gy)	$3B_{45}$	26.47	14.73	56.03	7.7	4	12.67	36.47	3.78	3	0.07	38	11.53	4
	$3B_{60}$	32.3	14.39	47.24	5.45	3	16	44	3.8	4	0.93	37.63	5.32	3
	$4B_{120}$	34.27	24.3	49.33	5.01	2	26.77	50.57	3.6	2	0.97	38.09	3.82	1
	5B	34.62	26.32	48.42	4.57	1	26.32	44.92	3.01	1	0.58	38.92	4.3	2
CHIASM D_{max}(Gy)	$3B_{45}$	29.45	23.11	53.5	9.33	1	15.55	44.05	5.56	4	1e-7	28.39	-1.07	3
	$3B_{60}$	27.1	23.23	51.1	10.31	2	14.8	41	4.96	2	1e-8	26.31	-0.8	2
	$4B_{120}$	26.06	22.68	50.93	11.29	4	14.86	41.06	5.22	3	1e-8	25.46	-0.6	1
	5B	27.92	25.06	51.89	10.53	3	16.02	41.32	4.76	1	1e-8	29.22	1.3	4
OPTIC_NERVE_L D_{max}(Gy)	$3B_{45}$	20.4	9.17	48.61	4.78	2	8.7	34.9	4.94	4	0.38	21.33	0.93	3
	$3B_{60}$	23.6	11.56	49.63	4.78	1	11.1	38	4.75	2	0.62	24.1	0.51	2
	$4B_{120}$	25.02	12.81	50.15	5.11	4	12.42	39.02	4.87	3	0.35	25.28	0.26	1
	5B	24.76	13.03	50.04	5.04	3	12.76	38.56	4.58	1	0.7	26.13	1.38	4
OPTIC_NERVEV_R D_{max}(Gy)	$3B_{45}$	23.48	9.68	48.61	6.89	4	9.98	40.68	5.9	4	0.3	27.27	3.8	4
	$3B_{60}$	30.18	14.63	52.59	6.6	1	14.78	45.98	5.54	2	0.65	32.46	2.29	2
	$4B_{120}$	29.82	14.47	53.26	6.77	3	14.62	45.42	5.61	3	0.25	32.08	2.26	1
	5B	31.78	16.37	53.09	6.67	2	16.58	46.38	5.23	1	0.67	34.58	2.81	3

TABLE D1: Continued

ROI/Matrix	Plan	sNRV+R Evaluation					Conventional Evaluation				p-value	Gold standard evaluation		
		Nominal	Min	Max	WD	RP	Minmum	Maximum	WD	RP		Accu_{Nom}	ΔD_{st}	RP
patient 3														
Low-risk-CTV D95(%)	$3B_{60}$	98.57	95.4	98.57	1.26	3	95.56	98.57	0.66	2	1e-5	98.25	-0.32	3
	$4B_{120}$	98.89	95.4	98.89	1.15	1	95.4	98.89	0.52	1	1e-4	98.89	0	1
	5B	98.57	95.08	98.57	1.17	2	94.76	98.57	0.69	3	0.01	98.57	0	2
CTV-T D95(%)	$3B_{60}$	98.48	91.87	98.48	1.21	1	92.7	98.48	0.75	2	0.01	98.21	-0.28	2
	$4B_{120}$	98.48	89.12	98.48	1.31	2	90.91	98.48	0.82	3	0.02	98.21	-0.28	2
	5B	98.48	91.87	98.48	1.33	3	93.39	98.48	0.6	1	1e-4	98.21	-0.28	2
CTV-T D2(Gy)	$3B_{60}$	103.72	102.89	106.75	0.6	3	102.75	104.68	0.24	2	1e-3	103.72	0	1
	$4B_{120}$	103.44	102.62	105.37	0.52	2	102.75	104.41	0.35	3	0.13	102.89	-0.55	3
	5B	103.17	102.07	105.65	0.38	1	101.93	103.58	0.22	1	0.01	102.89	-0.28	2
PAROTID D_{mean}(Gy)	$3B_{60}$	30.15	27.49	40.84	2.12	2	27.16	34.09	1.09	1	1e-3	32.88	2.72	3
	$4B_{120}$	29.75	27.1	41.62	2.12	1	26.69	34.1	1.3	3	1e-3	30.63	0.88	1
	5B	28.84	26.1	40.29	2.25	3	25.55	33.1	1.26	2	1e-3	30.7	1.87	2
ORAL D_{mean}(Gy)	$3B_{60}$	8.36	4.7	12.33	1.23	3	4.5	12.44	1.33	3	0.88	7.79	-0.58	2
	$4B_{120}$	8.49	5.19	11.66	0.99	1	4.96	11.56	1.1	1	0.65	7.79	-0.7	3
	5B	7.31	4.03	10.91	1.12	2	3.83	10.87	1.17	2	0.93	6.76	-0.56	1
LARYNX D_{mean}(Gy)	$3B_{60}$	34.1	19.34	41.66	3.1	1	22.94	40.23	2.98	2	0.77	35.14	1.04	1
	$4B_{120}$	34.57	16.73	49.62	4.69	3	21.91	40.93	3.33	3	0.21	37.32	2.75	3
	5B	32.1	16.69	43.46	3.36	2	21.77	37.78	2.66	1	0.3	33.46	1.36	2
COCHLEAR D_{mean}(Gy)	$3B_{60}$	14.15	9.94	26.62	3.26	3	10.18	27.14	2.88	3	0.38	17.63	3.48	3
	$4B_{120}$	17.9	14.59	29.85	2.39	2	14.67	30.42	2.52	2	0.64	20.8	2.9	2
	5B	18.93	15.57	30.68	2.33	1	15.67	31.21	2.5	1	0.65	21.7	2.77	1
BRAINSTEM D_{max}(Gy)	$3B_{60}$	40.18	36.11	53.48	4.02	3	33.98	53.68	3.19	3	0.11	43.34	3.16	3
	$4B_{120}$	47.42	43.35	58.28	2.59	1	43.62	59.42	2.52	1	0.82	49.91	2.49	1
	5B	45.89	39.4	57.94	3.36	2	40.49	58.59	3.1	2	0.9	48.65	2.76	2
SPINAL D_{max}(Gy)	$3B_{60}$	29.75	23.39	41.59	3.68	3	26.35	37.75	2.45	3	0.68	32.05	2.3	3
	$4B_{120}$	44.02	39.09	50.1	1.53	1	41.62	50.12	1.33	1	0.65	46.01	1.99	1
	5B	33.37	26.57	40.97	2.21	2	29.97	40.67	2.02	2	0.66	35.47	2.1	2
CHIASM D_{max}(Gy)	$3B_{60}$	33.94	22.24	50.15	6.05	3	20.64	45.54	5	3	0.15	36.41	2.47	3
	$4B_{120}$	37.59	23.16	51.88	5.89	2	23.69	48.09	4.56	2	0.15	38.41	0.82	2
	5B	36.68	22.29	50.36	5.74	1	22.68	46.48	4.45	1	0.14	36.74	0.06	1
OPTIC_NERVE_L D_{max}(Gy)	$3B_{60}$	31.63	16.55	56.31	6.71	3	17.33	49.23	5.78	3	0.35	31.37	-0.26	1
	$4B_{120}$	31.84	18.14	53.26	5.48	1	18.64	46.44	4.94	1	0.55	30.94	-0.9	3
	5B	31.55	17.66	53.52	5.6	2	18.05	46.05	5.03	2	0.51	30.72	-0.83	2
OPTIC_NERVEV_R D_{max}(Gy)	$3B_{60}$	35.31	20.44	56.31	7.12	3	19.71	53.51	5.93	3	0.38	34.85	-0.46	1
	$4B_{120}$	31.84	18.14	53.26	5.82	1	18.74	43.84	4.47	1	0.22	28.55	-3.29	3
	5B	31.55	17.66	53.52	6	2	18.25	44.05	4.58	2	0.2	28.48	-3.07	2
patient 4														
Low-risk-CTV D95(%)	$3B_{60}$	98.57	96.03	98.25	1.04	1	97.62	98.57	0.33	3	1e-8	97.94	-0.63	2
	$4B_{120}$	98.57	96.35	98.25	1.04	2	97.78	98.57	0.2	1	1e-13	98.25	-0.32	1
	5B	98.89	96.03	98.57	1.18	3	98.1	98.89	0.22	2	1e-15	98.25	-0.63	3
CTV-T D95(%)	$3B_{60}$	97.93	94.08	97.93	0.83	1	96.69	97.93	0.45	2	1e-3	97.38	-0.55	3
	$4B_{120}$	97.93	95.18	97.38	1.1	2	96.83	97.8	0.47	3	1e-9	97.66	-0.28	2
	5B	97.93	95.45	97.38	1.14	3	97.11	97.93	0.3	1	1e-13	97.93	0	1
CTV-T D2(Gy)	$3B_{60}$	103.44	102.89	103.99	0.13	1	103.31	104.13	0.16	1	1e-3	102.62	-0.83	3
	$4B_{120}$	102.89	102.89	103.72	0.37	3	102.75	103.72	0.18	2	0.01	103.17	0.28	2
	5B	102.89	102.62	103.72	0.25	2	102.89	103.72	0.26	3	0.85	102.89	0	1
PAROTID D_{mean}(Gy)	$3B_{60}$	28.1	24.17	38.89	2.82	1	24.49	32.12	1.41	1	1e-3	32.27	4.17	1
	$4B_{120}$	26.39	23.87	38.06	3.06	3	22.35	30.73	1.57	3	1e-4	31.27	4.88	2
	5B	26.27	23.86	37.73	3.01	2	22.38	30.42	1.48	2	1e-4	31.19	4.93	3
ORAL D_{mean}(Gy)	$3B_{60}$	17.65	14.44	23.25	1.28	1	15.69	21.34	1.01	3	0.17	18.63	0.98	3
	$4B_{120}$	16.77	13.7	20.58	1.57	3	14.92	20.47	1	2	0.11	17.69	0.93	2
	5B	16.67	13.63	20.42	1.51	2	14.78	20.36	0.97	1	0.11	17.56	0.89	1
LARYNX D_{mean}(Gy)	$3B_{60}$	30.92	26.59	36.07	1.89	1	28.95	34.59	1.04	1	0.9	31.57	0.64	1
	$4B_{120}$	32.33	24.09	41.39	3.01	3	28.8	39.73	2.1	3	0.12	36.49	4.16	3
	5B	28.8	22.69	34.62	2.2	2	26.51	33.41	1.24	2	0.34	31.06	2.27	2
COCHLEAR D_{mean}(Gy)	$3B_{60}$	18.75	15.33	28.42	2.09	1	14.04	25.52	2.33	3	0.66	18.91	0.16	1
	$4B_{120}$	20.49	17.18	27.62	2.31	3	17.04	26.75	2.11	2	0.98	20.78	0.29	2
	5B	21.13	17.98	28.24	2.23	2	17.9	27.23	2.05	1	0.89	21.46	0.33	3
BRAINSTEM D_{max}(Gy)	$3B_{60}$	39.62	36.43	52.81	5.98	3	29.22	46.82	3.55	2	1e-6	38.38	-1.24	1
	$4B_{120}$	37.12	28.64	53.18	5.13	2	26.82	46.12	3.6	3	0.09	35.04	-2.08	3
	5B	37.59	30.04	52.92	4.85	1	27.89	45.09	3.28	1	0.04	35.68	-1.91	2
SPINAL D_{max}(Gy)	$3B_{60}$	32.66	18.37	43.91	4.96	1	23.86	42.56	3.44	1	0.22	40.04	7.38	3
	$4B_{120}$	27.37	18.84	41.85	5.13	2	19.47	37.97	3.54	3	0.1	33.59	6.23	1
	5B	28.22	19.74	43.03	5.26	3	20.42	38.82	3.45	2	0.09	35	6.78	2
CHIASM D_{max}(Gy)	$3B_{60}$	37.02	39.55	59.74	16.08	3	24.92	47.62	4.1	3	1e-21	38.31	1.29	1
	$4B_{120}$	34.27	34.81	56.98	14	2	25.17	44.87	3.98	1	1e-17	37.08	2.81	3
	5B	35.45	34.6	56.7	13.66	1	24.85	45.85	4.03	2	1e-16	37.88	2.43	2
OPTIC_NERVE_L D_{max}(Gy)	$3B_{60}$	38.86	23.5	56.91	6.32	3	26.36	49.76	5.48	2	0.86	38.49	-0.37	1
	$4B_{120}$	40.87	27.07	54.72	5.54	1	27.67	51.77	5.33	1	0.82	39.42	-1.45	2
	5B	39.68	26.87	54.36	5.7	2	25.88	50.98	5.52	3	0.91	38.21	-1.47	3
OPTIC_NERVEV_R D_{max}(Gy)	$3B_{60}$	38.86	23.5	59.41	6.68	3	25.76	51.76	5.04	3	0.34	37.62	-1.25	2
	$4B_{120}$	40.87	29.48	54.72	5.29	1	27.87	51.47	4.57	1	0.61	38.55	-2.32	3
	5B	39.68	27.49	54.36	5.56	2	26.58	50.58	4.72	2	0.63	38.67	-1.01	1

Appendix E: Further evaluation with range uncertainty on one patient.

To further demonstrate that including range uncertainty is not likely to change the conclusion, we ran the evaluation with range uncertainty on patient 2, who experienced 10% weight loss during the treatment.

Three beam arrangements ($3B_{60}$, $4B_{120}$, 5B) of patient 2 were used to do the evaluation. In the sNRV+R evaluation, 35 (sNRV + rigid setup uncertainty) $\times 3$ (0, 3.5% and -3.5% range uncertainty) = 105 scenarios were generated. The 105 dose distributions for each IMPT plan were included in this sNRV+R evaluation.

In the conventional evaluation, the same 35 rigid setup uncertainty $\times 3$ (0, 3.5% and -3.5% range uncertainty) = 105 scenarios were used to calculate dose distribution.

The WD is used in robustness ranking for each dose metric. We compared the consistency of robustness ranking with and without range uncertainty. In the robust evaluation comparison with range uncertainty, $P_{C \leq 0}$ is 90%, 6.7% higher than the robust evaluation comparison without range uncertainty. It could be that in the gold standard evaluation, the Hounsfield Unit of weekly CT can be affected by noise and reflected as range uncertainty. With the range uncertainty, the sNRV+R evaluation is closer to the gold standard evaluation. To fully evaluate the plan, the range uncertainty should be used with the translation rigid setup and sNRVs.
